# Emergence of a Resource Acquisition Trade‐off at the Community Scale during Environmental Change

**DOI:** 10.1111/ele.70097

**Published:** 2025-04-01

**Authors:** Anton Pranger, Frank Peeters, Nathalie Wagner, Sebastian Diehl, Dietmar Straile

**Affiliations:** ^1^ Limnological institute University of Konstanz Konstanz Germany; ^2^ Integrated Science Lab—IceLab Umeå University Sweden

**Keywords:** community, community ecology, eutrophication, phosphate, phytoplankton, trade‐off, trait‐based ecology, traits, trophic state

## Abstract

Biomass‐weighted mean traits of a community's constituent species are a useful tool to assess environmental filtering in community function in response to environmental change. We show that annually averaged phytoplankton community function, expressed by the community mean traits phosphate and light affinity, responded strongly and reversibly to long‐term changes in nutrient supply over a 42‐year period of eutrophication and re‐oligotrophication of Lake Constance. Within the lake's species pool, phosphate and light affinities were weakly negatively correlated, suggesting a weak physiological trade‐off. Yet, a strong trade‐off between these traits emerged when species were weighted by their biomass, suggesting species sorting along the trade‐off line across years of shifting nutrient status. Emergent trade‐offs, that is, trade‐offs that become apparent first when trait combinations are weighted by the contributions of the trait‐bearing organisms to community biomass, may be a useful, novel concept in trait‐based ecology of potentially similar importance as commonly considered physiological trade‐offs.

## Graphical abstract

A four‐decadal cycle of eutrophication and subsequent re‐oligotrophication in Lake Constance led to the emergence of a strong trade‐off between phosphate and light affinity. We derive an explanation for the emergence of this trade‐off from resource ratio theory and discuss the relevance of this novel concept for trait‐based ecology.

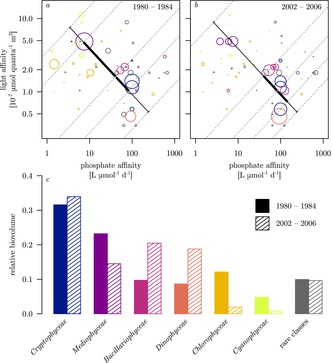

## 1 Introduction

Trait‐based ecology connects changes in community composition with changes in ecosystem function and provides a framework for understanding and predicting the effects of environmental variation on ecosystem dynamics (McGill et al. 2006). The traits of an organism determine the environment in which it is most successful. Directional changes in environmental conditions can thus result in patterns of succession driven by the traits of the community members. Frequently, such community changes expose trade‐offs between functional traits, of which at least one trait is directly affected by the environmental change (Litchman and Klausmeier 2008). Studies of succession have proposed several types of trade‐offs. For example, a decrease in rainfall favoured dry forest tree species with high wood densities over species with large leaf areas, suggesting a trade‐off between competition for water and maximum growth rate (gleaner–opportunist trade‐off, Lasky et al. 2016); a seasonal increase in grazer density coincided with a switch from fast‐growing to well‐defended phytoplankton, suggesting a growth rate–defence trade‐off (Ehrlich et al. 2020); and successional patterns on abandoned fields revealed a gradual replacement of seed‐heavy prairie grasses by strongly rooted ones followed by woody plants, suggesting a three‐way trade‐off between colonisation ability, nutrient competition, and competition for light (Tilman 1990).

According to the mass ratio hypothesis (Grime 1998), ecosystem functions are mainly determined by the traits of species that contribute most to community biomass. Conversely, the traits of the species that dominate community biomass may yield insights into the factors governing community composition. Consequently, community mean traits, determined as a biomass‐weighted mean of the traits of a community's constituent species, are expected to provide information about process rates in ecosystems and about the forcing factors affecting the community (Kleyer et al. 2012; McGill et al. 2006). Accordingly, community mean traits have been used to study the importance of environmental and/or biotic filtering in numerous types of communities including plants, bees, soil microbes, and phytoplankton (Bruelheide et al. 2018; Ricotta and Moretti 2011; Piton et al. 2020; Wentzky et al. 2020). In many of these studies, community mean traits have been used to identify trait–environment correlations along either spatial or temporal environmental gradients.

Because of short generation times and high trait diversity (Litchman and Klausmeier 2008), phytoplankton are particularly suited for the study of temporal trait–environment relationships. In many temperate, seasonally stratifying waters, environmental conditions vary strongly over the year and give rise to distinct patterns of seasonal succession in the functional composition of phytoplankton communities (Sommer et al. 1986). A majority of studies of trait–environment relationships in phytoplankton have therefore focused on the within‐year time scale and have documented seasonally recurrent changes in community mean traits such as specific growth rate, grazing resistance, and light/nutrient acquisition traits (Weithoff et al. 2015; Edwards et al. 2016; Ehrlich et al. 2020; Wentzky et al. 2020). Yet, many aquatic systems have also undergone environmental changes over longer, often decadal, time scales, a prime example being cultural eutrophication (Smith 1998). While trophic state has long been identified as a main axis in the environmental classification of phytoplankton (Willén 2000; Reynolds et al. 2002), phytoplankton trait responses to long‐term changes in nutrient availability have rarely been quantified, possibly because the high seasonal variation in phytoplankton dynamics requires long time series to make trait responses to longer term environmental changes detectable.

**FIGURE 1:**
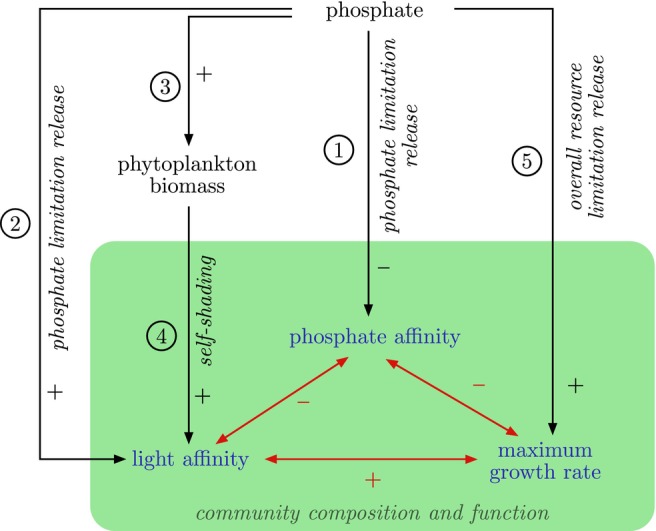
Hypothesised causal and correlational pathways by which a lake's nutrient state is related to the growth and resource acquisition traits of its phytoplankton community, and potential trait–trait relationships. An increase in phosphate is expected to directly decrease selection for phosphate affinity (arrow 1) and indirectly increase selection for light affinity (arrow 2) by releasing phosphate limitation and increasing the relative selective pressure of light limitation; to increase phytoplankton biomass and self‐shading and, thus, selection for light affinity (arrow 3 and 4); and to release overall resource limitation and, thus, increase selection for maximum growth rate (arrow 5). Single‐headed black arrows indicate causal directional relationships and double‐headed red arrows indicate hypothesised trait correlations. The green shaded area marks nodes that together constitute community composition and function. Expected correlations between nodes in the graph can be deduced by multiplying the signs along the paths linking them. In this work, we use total phosphorus during winter mixis (TP_mix_), as a proxy for the phosphate budget available to phytoplankton and an indicator of nutrient status. Additionally, we use biovolume as a proxy for biomass.

Here we report long‐term changes in mean traits of the phytoplankton community of deep, temperate Lake Constance over four decades of pronounced changes in external nutrient supply. During the study period, total phosphorus concentration (TP) varied 10‐fold, and phytoplankton community composition changed markedly (Jochimsen et al. 2013; Milan et al. 2022). We expected that changing nutrient supply would affect phytoplankton growth in three ways: (1) increased TP would result in release from phosphorus limitation (Figure 1, arrows 1 and 2); (2) since externally supplied solar radiation did not differ systematically over the study period, increased TP would result in reduced overall resource limitation and increased biomass (Figure 1, arrows 5 and 3); (3) increased biomass would result in more self‐shading and thus light limitation in periods of high nutrient availability (Figure 1, arrow 4).

Given these expectations, we hypothesised that changes in nutrient supply should have affected the phytoplankton response traits phosphate affinity, light affinity, and maximum growth rate: periods of higher TP should have selected for lower phosphate and higher light affinity in the phytoplankton community, yielding a negative correlation between community mean light and phosphate affinities across study years. Additionally, we hypothesise that periods of higher TP increased the relative benefit of high maximum growth rates, yielding a negative correlation between community mean phosphate affinity and maximum growth rate (i.e., a gleaner–opportunist trade‐off) and a positive correlation between community mean maximum growth rate and light affinity (Figure 1). Note that the hypotheses in Figure 1 are based on the assumption of a purely autotrophic mode of nutrition and may not apply to mixotrophic phytoplankton (which partially sustain resource needs heterotrophically). Given that mixotrophs were abundant in some study years, we also explored whether trait–environment and trait–trait relationships were sensitive to the exclusion of mixotrophic taxa.

## 2 Materials and Methods

### 2.1 Study system and calculation of biovolumes

**FIGURE 2:**
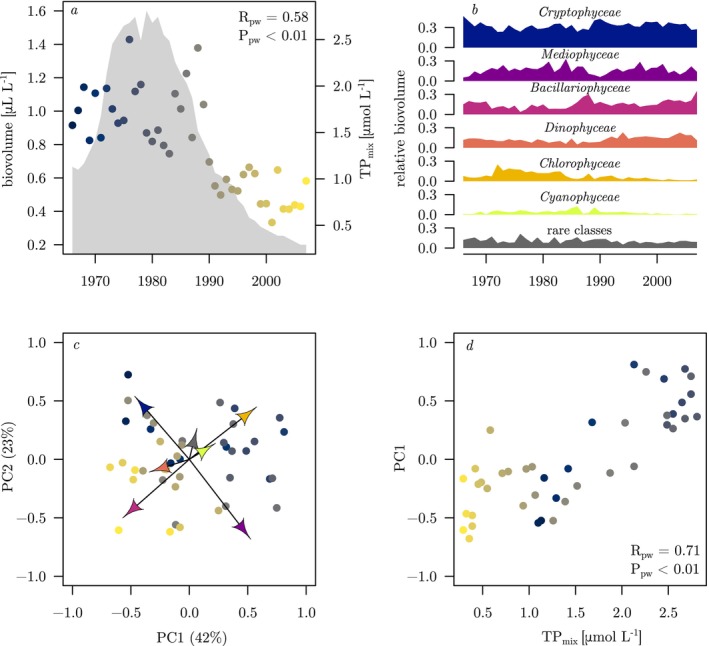
Lake Constance time series of (a) annual mean total phytoplankton biovolume (dots) and total phosphorus concentration during winter mixis (TP_mix_, grey shading) and (b) the relative biovolumes of the six most dominant taxonomic classes and the rare classes summed together. (c) Principal component analysis biplot of the relative biovolumes of the 27 classes. (d) Scores of principal component axis 1 (PC1) plotted against TP_mix_. Data points in a, c and d are coloured by year, from dark blue in 1966 to yellow in 2007. Panels a and d also show correlation coefficients (R_pw_) and probabilities (P_pw_) resulting from Prais‐Winsten regression of biovolume and PC1, respectively, against TP_mix_. The arrows in c are projections of the eigenvectors of the six taxonomic classes along the first two components, and the colour of the arrowheads indicates the groups as depicted in b. The eigenvectors of the rare classes are summed together, and the scores of PC1 and PC2 are scaled by a factor of 5 in order to depict them on the same axes as the eigenvectors.

Lake Constance, a large (area 536 km^2^), deep (max. depth 250 m), monomictic and temperate lake, has been the subject of over a century of limnological investigation (Straile 2021). Referenced against many other lakes, Lake Constance data have been instrumental in the development of ecological theory, including physical controls on phytoplankton and zooplankton blooms (Straile et al. 2012; Gronchi et al. 2021) and models of seasonal plankton succession (Sommer et al. 1986; Sommer et al. 2012). From the 1950s to 1980, the lake underwent a period of severe eutrophication, followed by several decades of re‐oligotrophication due to the construction of wastewater treatment facilities. Phosphorus was the main driver of trophic change (Jochimsen et al. 2013; Gaedke and Schweizer 1993; Milan et al. 2022), peaking in 1979 at an annual average TP value of 2.8 μmol L^−1^ and declining to 0.39 μmol L^−1^ by the mid‐2000s (Güde and Straile 2016; Figure 2a). Other potentially growth‐limiting variables (mineral nitrogen, silicon and light) remained stable during oligotrophication (Jochimsen et al. 2014; Güde and Straile 2016).

Here, we follow Jochimsen et al. (2013) and use total phosphorus concentration during winter mixis (TP_mix_) at the central monitoring station of Upper Lake Constance as an indicator of the lake's nutrient state during a 42‐year study period from 1966 to 2007. During this period, TP_mix_ changed by approximately an order of magnitude: it increased roughly threefold from the mid‐1960s until the end of the 1970s, and subsequently declined to roughly a tenth of peak values (Figure 2a).

During the study period, integrated water samples covering the depth range 0–50 m from 1966–1976 and afterwards from 0 to 20 m were taken at approximately biweekly intervals at station Fischbach‐Uttwill. Phytoplankton in these samples were microscopically identified and counted, and cell volumes were determined to obtain estimates of biovolume $B_{i}\left(t\right)$ of each species *i* on sampling date *t* (IGKB, ISF), which we use as proxies for each species' wet biomass. Biovolumes from 1966 to 1976 were renormalised to a 0–20 m depth range using methods described in Jochimsen et al. (2013), which contains a more detailed description of phytoplankton sampling and biovolume estimation.

From the species‐specific biovolumes, we calculated three variables for each sampling date: (1) *community biovolume* summed over all *n* species, $B\left(t\right)=\sum_{i=1}^{n}B_{i}\left(t\right)$; (2) the *relative biovolume of each species*, $b_{i}\left(t\right)=B_{i}\left(t\right)/B\left(t\right)$; and (3) the *relative biovolumes of taxonomic classes*, $g_{j}\left(t\right)=G_{j}\left(t\right)/B\left(t\right)$, where $G_{j}\left(t\right)=\sum_{i\in I_{j}}B_{i}\left(t\right)$ and $I_{j}$ is the set of indices i corresponding to species belonging to class *j*. For each of the 42 study years y, we then calculated the annual means of these three variables, $B\left(y\right)$, $b_{i}\left(y\right)$, and $g_{j}\left(y\right)$, by linearly interpolating between sampling days and subsequently averaging over the 365 or 366 days of the respective year. Calculated that way, the annual mean relative biovolumes $b_{i}\left(y\right)$ and $g_{j}\left(y\right)$ reflect the average composition of the community without being dominated by spring and summer blooming taxa, and emphasise the response of the phytoplankton community to long‐term changes in nutrient supply. For each species *i*, we also calculated average relative biovolume across the entire 42‐year period as $\bar{b_{i}}=\frac{1}{42}\sum_{y=1966}^{y=2007}b_{i}\left(y\right)$.

Due to developments in microscopy, small phytoplankton were less accurately identified in the earlier sampling years. To avoid bias due to identification errors, we excluded taxa with cell volumes < 30 μm^3^ from the analysis of community traits. We thus excluded 7.5% of the observed taxa, which contributed on average 1.8% to the annual mean community biovolume. The remaining taxa belonged to 27 different classes. In figures, we present only the six most dominant classes, that is, *Cryptophyceae*, *Mediophyceae* (polar centric diatoms), *Bacillariophyceae* (pennate diatoms), *Dinophyceae*, *Chlorophyceae*, and *Cyanophyceae*. These classes contributed on average 89% (range: 79%–95%) to the annual mean community biovolume (summed over all taxa with cell volumes ≥ 30 μm^3^). The remaining classes are grouped together and labelled ‘rare classes’.

### 2.2 Determination of Species and Community Traits

We focus on traits that we suspected to be responsive to external nutrient supply, that is, the resource acquisition traits phosphate and light affinity, and the functional trait maximum growth rate (Figure 1). These traits are parameters of Monod equations (Monod 1949) describing specific growth rates under phosphorus and light limitation, respectively:

(1) $$\mu_{P}=\mu_{\max}∙\frac{P}{\frac{\mu_{\max}}{\gamma }+P}$$

(2) $$\mu_{I}=\mu_{\max}∙\frac{I}{\frac{\mu_{\max}}{\alpha }+I}$$

Here, $\mu_{P}$ (d^−1^) and $\mu_{I}$ (d^−1^) are the specific growth rates of phytoplankton under phosphate and light limitation, respectively, $\mu_{\max}$ (d^−1^) is the maximum growth rate, γ is phosphate affinity (L μmol^−1^ d^−1^), P is phosphate concentration (μmol L^−1^), α is light affinity (μmol quanta^−1^ m^2^) and I is light intensity (μmol quanta m^−2^ d^−1^). Note that the half saturation constants for phosphate *k*_*P*_ and light *k*_*I*_ often used with the Monod equation are equal to the maximum growth rate–affinity ratio, that is, $k_{P}=\mu_{\max}/\gamma$ and $k_{I}=\mu_{\max}/\alpha$.

We assigned values for the above three traits to the phytoplankton species in Lake Constance using published data from controlled resource limitation experiments to which parameters of the Monod equation had been fitted (Bruggeman 2011; Edwards et al. 2013; Schwaderer et al. 2011). Since the published datasets covered only parts of the Lake Constance species pool, missing trait values were interpolated based on taxonomy retrieved from Algaebase.org (Guiry and Guiry 2021). A missing trait value of a species was estimated as the geometric mean of the trait values of its closest relatives for which measured data were available. A detailed method description and sensitivity analysis of missing trait imputation are provided in SI 1. Taxonomy and trait estimates are provided in Tables [Link], [Link].

Since we were interested in trait responses to long‐term environmental changes, we excluded seasonal trends from our analyses and focused exclusively on annually averaged community composition. Because of the logarithmic distribution of traits, we calculated community mean traits as geometric means of the traits of all species weighted by their annual mean relative biovolumes. Traits were log‐transformed prior to weighting, yielding

(3) $$\mathrm{CMT}\;\left(y\right)=\exp\left(\sum_{i=1}^{n}\ln\left(\tau_{i}\right)\cdot b_{i}\left(y\right)\right)$$

where $\mathrm{CMT}\left(y\right)$ is the value of the community mean trait in year y, $\tau_{i}$ is the trait value of species i and $b_{i}\left(y\right)$ is the annual mean relative biovolume of species i in year y (see section *Study system and calculation of biovolumes)*. In addition to community mean traits, we calculated group mean traits of the six dominant taxonomic classes and tested their relationship with TP_mix_. Methods and results of this analysis are shown in SI 1.

### 2.3 Statistical Analyses

To investigate whether changing nutrient supply affected the taxonomic and functional composition of the phytoplankton community, we performed a principal component analysis (PCA) on annual mean relative biovolumes of the 27 taxonomic classes $g_{j}\left(y\right)$ across the 42 study years and subsequently tested for correlation between the first principal component (PC1) and TP_mix_. To explore, whether changes in TP_mix_ were associated with systematic trait changes through mechanisms hypothesised in Figure 1, we tested whether community mean traits $\mathrm{CMT}\left(y\right)$ were correlated to TP_mix_ and PC1, and whether phytoplankton biovolume was correlated with both TP_mix_ and community mean light affinity. To account for temporal auto‐correlation in these relationships, we used the Prais–Winsten method (Bence 1995, R package prais version 1.1.3), which corrects regression statistics for type‐1 auto‐correlation. Correlation coefficients and probabilities obtained through this method are denoted R_pw_ and P_pw_.

We performed standardised major axis (SMA) fitting to search for statistical associations between the investigated traits at three levels of ecological and temporal integration. (1) We first obtained the SMAs of unique trait pairs, that is, phenotypes, belonging to species found in Lake Constance, where all phenotypes were given an equal weight. (2) We then repeated the same analysis, but with each phenotype's traits being weighted by the average relative biovolume of species with the respective phenotype across the entire 42‐year period $\bar{b_{i}}$. (3) Finally, we obtained SMAs of the annually averaged community mean traits across the 42 study years *CMT(y)*. A detailed explanation of SMA fitting techniques is found in SI 3. Pearson correlation coefficients and probabilities corresponding to SMA are denoted R and P.

The establishment of trait–environment and trait–trait associations based on parametric approaches involving community mean traits has been criticised on statistical grounds, and additional approaches have been proposed to assess their statistical significance (e.g., Peres‐Neto et al. 2017; Ter Braak et al. 2018). We therefore complemented the parametric approaches described above with permutation tests. In each permutation, we paired all 42‐year time series of relative species biovolumes $b_{i}\left(y\right)$ with traits of randomly drawn species (without replacement) from the Lake Constance species pool and recalculated the resulting 42‐year time series of community mean trait values CMT(*y, p*_*j*_) according to equation 3, where *p*_*j*_ indicates the *j* ^th^ out of *k* permutations. For each permutation *j*, we calculated how much variance of the permutated community traits CMT(*y, p*_*j*_) was explained by TP_mix_ or biovolume (using R_pw_^2^), respectively, or by correlations between trait pairs (phosphate vs. light affinity, phosphate affinity vs. maximum growth rate, light affinity vs. maximum growth rate, using R^2^). We performed *k* = 10^6^ independent permutations and used the fraction of permutations with explained variance larger than that in the original correlations as an estimate of the probability that an observed trait–TP_mix_, trait–biovolume or trait–trait association could have arisen by chance.

To examine whether trait–environment and trait–trait relationships were sensitive to the exclusion of mixotrophs, we performed all of the above analyses (except for permutation tests) on data excluding potentially mixotrophic taxa. These analyses revealed that some outcomes were sensitive to the exclusion of *Dinophyceae*, but not to the exclusion of other potentially mixotrophic taxa (Figure S8). We therefore only report detailed results from the analyses excluding *Dinophyceae*. Methods to calculate community mean traits excluding *Dinophyceae* are described in SI 2. All statistical analyses and visualisations of results were performed with R (version 4.4.1).

## 3 Results

Over the 42‐year study period, total phytoplankton biovolume was positively correlated with TP_mix_ (Figure 2a). Based on annual average relative biovolumes (Figure 2b), *Cryptophyceae* were the most dominant class (mean 33%, range: 23%–47%), followed by *Mediophyceae* (mean: 17%, range: 5.3%–33%), *Bacillariophyceae* (mean: 16%, range: 3.7%–35%), *Dinophyceae* (mean: 13%, range: 4.8%–23%), *Chlorophyceae* (mean: 7.9%, range: 1.4%–25%) and *Cyanophyceae* (mean: 3.5%, range: 0.20%–12%). The contribution of rare classes was on average 11% (range: 5.1%–21%). Correlations of relative class biovolumes against TP_mix_ (Figure S2) were negative for *Cryptophyceae*, *Bacillariophyceae*, and *Dinophyceae* (R_pw_ = −0.55, R_pw_ = −0.45 and R_pw_ = −0.62, respectively), positive for *Chlorophyceae* and *Cyanophyceae* (R_pw_ = 0.70 and R_pw_ = 0.48, respectively) and absent for *Mediophyceae*.

The first axis of the PCA explained 42% of inter‐annual variability in the relative class biovolume (Figure 2c). The relative biovolumes of *Mediophyceae*, *Chlorophyceae*, and *Cyanophyceae* had a positive eigenvector component along the first principal component axis (PC1), while *Bacillariophyceae*, *Cryptophyceae*, and *Dinophyceae* had a negative component. Along the second principal component axis (PC2, 23% explained variance), *Cryptophyceae*, *Chlorophyceae*, and *Cyanophyceae* had a positive component, and the components of *Mediophyceae*, *Bacillariophyceae*, and *Dinophyceae* were negative. The sum of the eigenvectors of the rare classes was positive along both principal component axes. While scores of PC1 were tightly correlated with TP_mix_ (Figure 2d), PC2 and TP_mix_ were not significantly correlated (R_pw_ = 0.25, P_pw_ = 0.11). We therefore only focused on PC1 in subsequent analyses.

**FIGURE 3:**
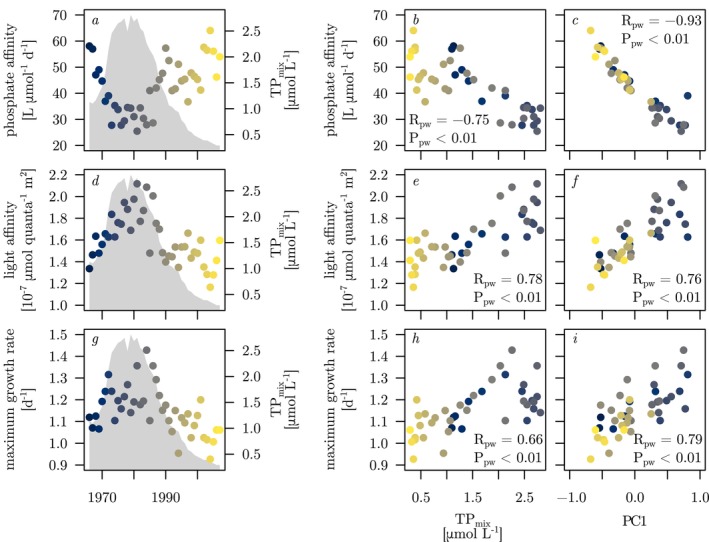
Community mean traits phosphate affinity (a–c), light affinity (d–f) and maximum growth rate (g–i) plotted against study year (left panels), TP_mix_ (middle panels) and the first principal component (PC1) from Figure 2c (right panels). Grey shading in the left panels indicates TP_mix_, and the colour of the data points indicates the sampling year, as in Figure 2a. Correlation coefficients (R_pw_) and probability values (P_pw_) result from Prais–Winsten regression of the traits against TP_mix_ (middle panels) and PC1 (right panels). PC1 scores are scaled by a factor of 5, as in Figure 2.

Community mean phosphate affinity varied over time in a way that was roughly opposite to the changes in TP_mix_ (Figure 3a). Consequently, community mean phosphate affinity was negatively correlated with both TP_mix_ and PC1 (Figure 3b–c). In contrast, community mean light affinity and maximum growth rate varied in approximate synchrony with the changes in TP_mix_, resulting in positive correlations with both TP_mix_ and PC1 (Figure 3d–i). Throughout, the correlations of the above three traits with PC1 and TP_mix_ were statistically highly significant, and the explained variances of trait relationships with PC1 were similar to or higher than those with TP_mix_. Community mean light affinity was also positively correlated with phytoplankton biovolume (data not shown, R_pw_ = 0.43, P_pw_ < 0.01).

P‐values computed with permutation tests were in line with those of parametric tests for the correlations of TP_mix_ with community mean phosphate affinity (*p* = 0.010) and community mean light affinity (*p* = 0.021). In contrast, the positive correlation of community mean maximum growth rate with TP_mix_ (Figure 3h) was not statistically significant in the permutation test (*p* = 0.43). Closer inspection suggested that exceptionally low maximum growth rates of *Dinophyceae* (see SE1, Figure 2) are the likely reason for this sensitivity to permutation. When these typically mixotrophic taxa were excluded from the calculation of community mean traits, relationships of recalculated community mean phosphate and light affinities with TP_mix_ and PC1 remained significant, but the correlation of community mean maximum growth rate with TP_mix_ broke down and its correlation with PC1 was reduced (Figure S5). The light affinity–biovolume relationship was insignificant in the permutation test (*p* = 0.71).

**FIGURE 4:**
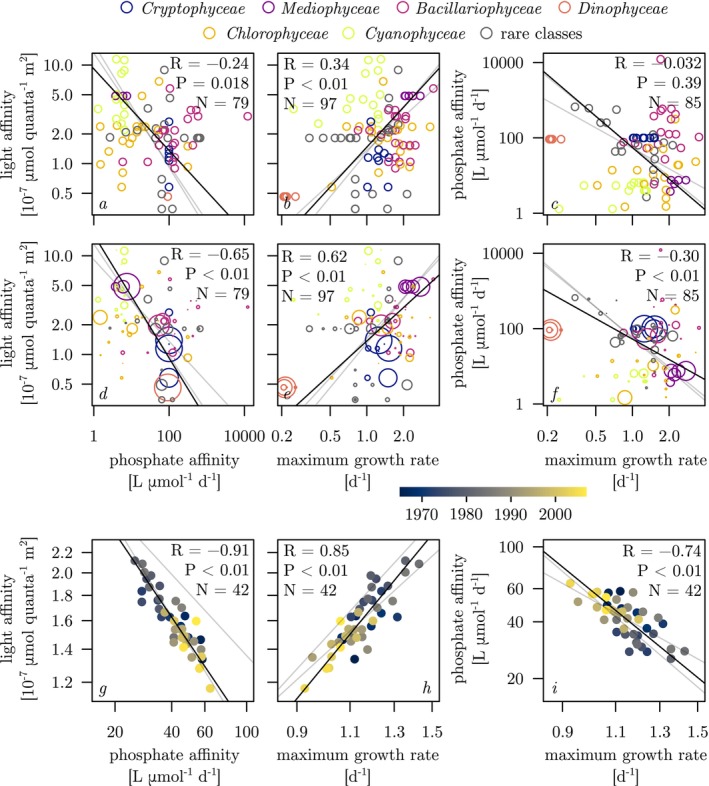
Pairwise plots of phosphate affinities, light affinities and maximum growth rates of Lake Constance phytoplankton phenotypes at three levels of ecological and temporal integration. (1) *Community membership* (panels a–c), where circles represent the unweighted values of unique trait pairs (= phenotypes) that were present in the community. (2) Biovolume‐weighted *average community composition* (panels d–f), which uses the same data, but the area of the circles is weighted by the respective 42‐year average relative biovolume corresponding to the unique trait pairs during the 42‐year study period, $\bar{b_{i}}$. (3) *Community mean traits* (panels g–i) for each of the 42 study years. SMA lines (in black) and statistical properties R and P were determined in SMA fittings on log‐transformed traits, the methods of which are described in SI 3. N indicates the number of unique trait pairs (a–f) or the number of years (g–i) used to determine P. To facilitate comparison of slopes and intercepts across the three levels of ecological and temporal integration, SMA from the two panels above and/or below are always shown, but with increased transparency. Values of slopes estimates and confidence intervals are shown in Figure 6a–c (SI 1). In panels a–f, open circles are coloured as in Figure 2b to indicate the taxonomic classes to which the different trait pairs belong. In panels g–i, closed circles are coloured as in Figure 2a to indicate the sampling year.

Correlation tests detected pairwise correlations between all three traits, the strength of which depended on the level of ecological and temporal integration expressed as *community membership*, biovolume‐weighted *average community composition* over the entire study period, and annually averaged *community mean traits*. At the community membership level, light affinity was negatively correlated with phosphate affinity (Figure 4a), and positively correlated with maximum growth rate (Figure 4b). The explained variances were, however, small (5.8% and 11%, respectively). Phosphate affinity was not significantly correlated with maximum growth rate (Figure 4c). However, at the average community composition level, when phenotype traits were weighted by average relative biovolumes across the 42‐year period, $\bar{b_{i}}$, significant relationships emerged between all three traits, with considerably higher explained variances (Figure 4d–f). Correlations were strongest at the level of community mean trait dynamics during the 42 study years. The explained variances where 83% and 55%, respectively, in the negative correlations of community mean phosphate affinity with community mean light affinity and growth rate, and 72% in the positive correlation between community mean light affinity and maximum growth rate (Figure 4g–i).

Permutation tests supported the statistical significance of observed trait–trait correlations at the level of community mean trait dynamics. Permutation‐derived P‐values were 0.004 for the negative correlation between community mean phosphate and light affinities, 0.044 for the positive correlation between community mean maximum growth rate and light affinity, and 0.048 for the negative correlation between community mean maximum growth rate and phosphate affinity.

When *Dinophyceae* spp. were excluded from community mean trait calculations, SMA slopes and explained variance remained unchanged for the negative correlations between phosphate and light affinity at all three levels of ecological and temporal integration (Figure S6 and S7). Correlations involving maximum growth rate, however, were no longer significant at the unweighted species level but remained significant at higher levels of ecological and temporal integration, albeit with considerably lower fractions of variance explained (Figure S6 and S7).

## 4 Discussion

Lake Constance underwent drastic changes in trophic state over a period of four decades covering an almost complete cycle of intense eutrophication and subsequent re‐oligotrophication. The phytoplankton community, described by annually averaged taxonomic composition, shifted systematically in response to changes in external nutrient supply as indicated by the strong correlation of PC1 and TP_mix_ (Figure 2d). Changes in community composition came with concomitant changes in community mean traits as indicated by the strong correlations of community mean phosphate affinity, light affinity, and maximum growth rate with PC1 (Figure 3 right panels). Phosphate and light affinity were, in turn, strongly negatively correlated, suggesting that changes in community composition in response to shifting nutrient supply were constrained by a trade‐off between these two resource acquisition traits. Remarkably, the phosphate–light affinity trade‐off was weak when analysed at the interspecific level across the species pool (Figure 4a) but emerged as very strong at the community scale (Figure 4g), suggesting species sorting along this trade‐off line across years of shifting nutrient status.

Our finding of a phosphate–light affinity trade‐off is in line with Litchman and Klausmeier's (2008) perception that environmental change can expose trade‐offs between functional traits if at least one trait is directly acted upon by the environmental change. In line with our hypotheses (Figure 1), the observed changes in the phytoplankton community suggest that phosphate affinity is the trait that was most directly affected by changes in nutrient supply. This hypothesis is supported statistically—of the investigated traits, phosphate affinity showed the strongest correlations with both TP_mix_ and PC1. More importantly, this hypothesis is in line with mechanistic expectations: all else being equal, lower phosphate availability in oligotrophic systems should make taxa with high phosphate affinity more competitive.

On first glance, the data are also in line with the hypothesis that eutrophication indirectly selected for higher light affinity by increasing phytoplankton biomass and self‐shading in years of higher nutrient availability (Figure 1). Intriguingly, however, the direct correlation between TP_mix_ and light affinity (61% explained variance) was stronger than correlations along the indirect path TPmix–biomass–light affinity (33% and 18% explained variance, respectively) and a permutation test suggested that the biomass–light affinity relationship was spurious. This indicates that the positive relationship between TP_mix_ and community mean light affinity cannot be explained by annually averaged phytoplankton biomass alone. The similarity of the SMA slopes between phosphate and light affinity at all three levels of ecological and temporal integration in Figure 4 is indeed compatible with the emergence of a community‐level trade‐off through two complementary mechanisms. First, the species pool already displays a weak trade‐off at the community membership level (Figure 4a). This trade‐off could be a consequence of selection on community membership in response to environmental conditions. However, as phytoplankton interact with their environment through a large number of traits, a weak trade‐off at the community membership level could also reflect physiological constraints in high‐dimensional trait space, meaning that increased allocation to one trait necessarily reduces allocation to others and that deviation from the two‐way trade‐off line may be explained by one or more unaccounted traits (Litchman and Klausmeier 2008).

Second, the affinity traits of the most abundant taxa were located closely to the SMA (Figure 4d). In the context of resource ratio theory, such a trait distribution would be predicted to occur in landscapes where the relative supply of two essential resources varies in space (Tilman 1986; Koffel et al. 2016; Wickman et al. 2019), but the concept is also applicable to temporal variation in resource ratios. The oligotrophic period should have had substantially reduced summer phosphate concentrations and therefore the ratio of light vs. phosphate availability and the nature of resource limitation changed substantially. Due to the nonlinear nature of resource limitation (Equation 1 and 2), the resource affinity of a species becomes exceedingly important at low resource levels. Consequently, species with high phosphate affinities will experience increased relative fitness at low phosphate concentrations. Conversely, under sufficiently high phosphate concentrations, light can become the limiting factor, and specific growth rates and fitness are less dependent on phosphate affinity, but predominantly on light affinity. Averaged over the year, the contribution of high light‐low phosphate affinity taxa to community mean traits should then increase with TP_mix_ and produce the observed trait–TP_mix_ and trait–trait correlations.

**FIGURE 5:**
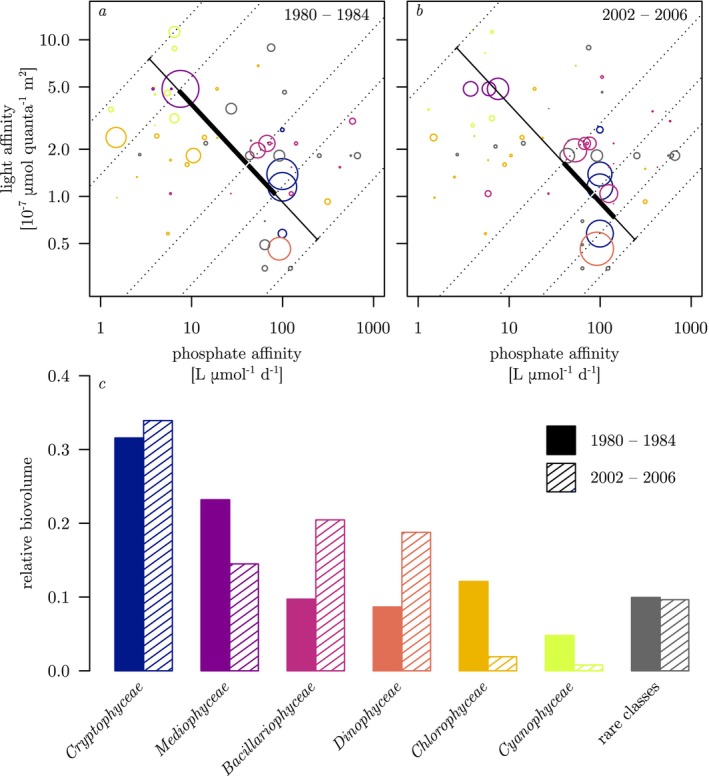
Average relative biovolume distribution in resource affinity trait space during (a) five years (1980–1984) with high nutrient availability (TP_mix_ > 2 μmol L^−1^) and (b) five years (2002–2006) with low nutrient availability (TP_mix_ < 0.4 μmol L^−1^). Each circle represents a unique trait combination (= phenotype), the circle area being proportional to the phenotype's relative biovolume averaged over the five‐year period. A phenotype can represent several species with identical traits, and its colour identifies the class to which the phenotype belongs (same colour code as in panel c). The black line in a and b corresponds to the weighted standardised major axis of log‐transformed phosphate affinity and light affinity in Figure 4d. Overlaid on this line is a boxplot, the whiskers, box range and bisecting line of which show the 0th and 100th, 25th and 75th and 50th percentiles, respectively, of standardised major axis scores weighted by the average relative biovolume from their respective period. The dotted lines perpendicular to the major axis identify the percentiles to which a given phenotype is counted. A detailed method description for calculating weighted percentiles on standardised major axis scores is presented in SI 3. Bar plots (c) depict the average relative biovolumes of the six most dominant classes and the rare classes during the five high nutrient years (solid fill) and the five low nutrient years (hatch fill).

Two lines of evidence support the above proposition. First, a comparison in phosphate–light affinity trait space of average community composition during five years near the peak of eutrophication (1980–1984) and near the far end of re‐oligotrophication (2002–2006), respectively, reveals that observed differences in community mean traits between these extreme trophic states were primarily driven by shifts in relative biovolumes along the SMA describing the emergent trade‐off (Figure 5). Specifically, typical summer taxa with low phosphate and high light affinity (such as most *Chlorophyceae* and *Cyanophyceae*) benefitted in high TP_mix_ years when summer phosphate limitation was reduced, whereas summer taxa with high phosphate and low light affinity (such as most *Dinophyceae* and *Bacillariophyceae*) benefitted in low TP_mix_ years when summer phosphate limitation was increased (Figure 5). Second, correlation tests of the relative abundances of the six most abundant phytoplankton classes against TP_mix_ uncover similar relationships across the entire study period, that is, strong positive correlations of TP_mix_ with *Chlorophyceae* and *Cyanophyceae*, and strong negative correlations of TP_mix_ with *Dinophyceae* and *Bacillariophyceae* (Figure S2).

In a trait‐based analysis of seasonal phytoplankton succession in Rappbode Reservoir, Wentzky et al. (2020) observed a weak anticorrelation between phosphate and light affinity at the level of community membership (approximately 12% explained variance), as well as opposing seasonal patterns of community mean phosphate vs. light affinity. These observations are in line with ours and therefore appear consistent with the emergence of a trade‐off between phosphate and light affinity also at the seasonal time scale. Yet, Wentzky et al. did not investigate this statistically and made no mention of such a trade‐off. It is therefore unclear how the seasonal pattern of the community mean phosphate and light affinity in their study compares quantitatively to the strong anticorrelation between these traits that we observed at inter‐annual time scales. A main novelty of our study compared to similar trait‐based analyses (Wentzky et al. 2020; Ehrlich et al. 2020) is that we made no a priori assumptions about physiological trait covariance and instead explore the realised functional changes resulting from a changing environment (SI 1).

Excluding mixotrophs from analyses revealed that trait–environment and trait–trait relationships involving maximum growth rate were somewhat sensitive to the exclusion of one class of mixotrophs, that is, *Dinophyceae* (Figure S5, and S6), but not of other potentially mixotrophic taxa such as *Chrysophyceae*, *Synurophyceae*, and *Coccolithophyceae* (Figure S8). In growth experiments, *Dinophyceae* typically grow remarkably slowly at rates below 0.5 d^−1^ (Chapman and Pfiester 1995; Bolch et al. 2017; Jakobsen et al. 2000) and are in this regard outliers in phytoplankton trait data. *Dinophyceae* mixotrophy comes in many forms—some *Dinophyceae* can form mutualistic interactions with bacterial communities that supply them with essential nutrients and vitamins, while others feed on algal or bacterial prey, and some can retain their prey's chloroplasts (Bolch et al. 2017, Jakobsen et al. 2000). Indeed, growth responses of *Dinophyceae* strongly depend on bacterial community composition and on availability and species of bacterial and algal prey (Bolch et al. 2017, Jakobsen et al. 2000). Some species may even die out in axenic cultures (Bolch et al. 2011). Because of these different modes of nutrition, a characterisation of mixotrophic *Dinophyceae* species in terms of Monod equation parameters may be inappropriate or even invalid. Consequently, the use of their Monod parameters in trait‐based studies may yield confounding patterns.

Since phosphate concentrations increased substantially, but incident radiation remained approximately constant, eutrophication did not only shift the primary resource limitation from phosphate to light, but presumably also reduced resource limitation overall, leading to the hypothesised gleaner–opportunist trade‐off (Figure 1). This hypothesis is only partially supported by the data. We indeed observed a negative correlation between phosphate affinity and maximum growth rate both at the biovolume‐weighted and community mean trait levels, regardless of whether *Dinophyceae* were removed from the analysis or not (Figure 4, Figure S6). However, the absence of a significant correlation between community mean maximum growth rate and TP_mix_ in the analysis excluding *Dinophyceae* (Figure S5h) suggests that shifting nutrient supply may not have been the main driver for this trade‐off.

Previous studies have shown that changes in the composition of the phytoplankton community of Lake Constance during eutrophication and subsequent re‐oligotrophication were reversible at both the species level (Milan et al. 2022) and the taxonomic class level (Jochimsen et al. 2013), which is also supported by the strong correlation between PC1 and TP_mix_ in our study. Our finding of an emerging trade‐off between phosphate affinity and light affinity at the community level suggests a mechanistic explanation for this reversibility in taxonomic community composition. Namely, when phosphate is restored to historical concentrations after a period of high phosphate loading, increased phosphate limitation should restore the contribution of high phosphate–low light affinity taxa that characterise an oligotrophic community. We are aware of only a few studies that have documented reversible changes in community composition over decadal time scales (Mittelbach et al. 2006; Matthews et al. 2013; Yuhara et al. 2023). Our study goes beyond this, that is, it addresses reversibility in community function quantified by community mean traits. The continuous distribution of the annually averaged mean phosphorus and light affinities along the TP_mix_ gradient suggests that community function changed smoothly and continuously in response to changes in nutrient supply during both the eutrophication and oligotrophication phases.

We conclude by highlighting the most important finding of our study, that is, the discovery of a strong emergent trait trade‐off at the community scale despite evidence for a physiological trade‐off being weak at best. Because physiological trade‐offs can exist in high‐dimensional trait space, this finding could have been easily overlooked if the distribution of phytoplankton biovolume had not been considered. By giving more weight to the more abundant taxa, the dimensionality of physiological trade‐offs is essentially reduced, and the realised trade‐off associated with the largest environmental gradient is exposed. We believe that this phenomenon might not be specific to our study and that our approach to trait‐based ecology may lead to the discovery of emergent trade‐offs in various types of ecological communities and in response to a wide range of environmental gradients. We propose that emergent trade‐offs are a useful, novel concept of potentially similar importance as the more commonly considered physiological trade‐offs.

## Author Contributions

N.W. and D.S. performed preliminary work. A.P. analysed the data, wrote the first draft of the manuscript, and produced figures. F.P., S.D., and D.S. provided substantial feedback, and A.P., F.P., S.D., and D.S. contributed substantially to the development of ideas, analyses, and revisions.

## Supporting information


Supplementary Information.

**Table S1.** Imputed phosphate affinities for phytoplankton taxa observed in Lake Constance obtained with methods described in S1.

**Table S2.** Imputed light affinities for phytoplankton taxa observed in Lake Constance obtained with methods described in S1.

**Table S3.** Imputed maximum growth rates for phytoplankton taxa observed in Lake Constance obtained with methods described in S1.

**Table S4.** Taxonomy of phytoplankton taxa observed in Lake Constance used for trait imputation.

## Data Availability

Lake Constance data was provided by the Institut für Seenforschung (ISF) and the Internationale Gewässerschutzkommission für den Bodensee (IGKB) from their Bodensee‐Wasser‐Informationssystem (https://www.igkb.org/daten‐und‐karten). Trait data were obtained from published data sets and phytoplankton taxonomy was downloaded from AlgaeBase. Annual mean data derived from Lake Constance data, trait data, taxonomy and code for reproducing statistical results and generating figures are publicly available at https://doi.org/10.5061/dryad.vx0k6dk2f.
