# Supplementary material for: Emergence of a Resource Acquisition Trade‐off at the Community Scale during Environmental Change
Source: Ecol Lett. 2025 Apr 1;28(4):e70097. doi: 10.1111/ele.70097 (PMC11959684; doi:10.1111/ele.70097)
Supplement: Supplementary file 1 — Supplementary Information. [file ELE-28-0-s002.pdf]

SI 1 – Trait imputation

We assigned phosphate affinities, light affinities and maximum growth rates to phytoplankton taxa observed in Lake Constance from trait data sets (Bruggeman 2011, Edwards et al. 2013, Schwaderer et al. 2011). These data include phosphate affinities of 38 taxa, light affinities of 53 taxa and maximum growth rates of 64 taxa. Direct matches between trait data sets and the 268 taxa observed in Lake Constance resulted in 22 phosphate affinity assignments, 28 light affinity assignments and 40 maximum growth rates assignments. Trait values of the remaining taxa were imputed based on the trait data sets and taxonomy obtained from AlgaeBase. When a trait value of an observed taxon was missing, we assigned the geometric mean of the trait values of taxa within the same genus. If no trait data were available at genus level, then the geometric mean of taxa within the same family was assigned. This step was repeated at successively higher taxonomic levels until all observed taxa were assigned a trait. Imputed traits and taxonomy are provided in tables S1-4.

This method preserves phylogenetic signals within the data across low and high taxonomic levels. Geometric means were used because of the logarithmic distribution of traits in the data sets. Within the six dominant classes, all traits were imputed at a taxonomic resolution below the class level, with the exception of phosphate affinities of the *Dinophyceae* spp., which were averaged over all *Chromista* spp. Over the study period, the taxonomic description and number of identifiable phytoplankton species has considerably increased, leading to instances where taxa were replaced by other taxa or split into two or more new taxa, resulting in an increase over time in the number of taxa found in Lake Constance (Jochimsen et al., 2014). These changes usually occurred at low taxonomic levels, and therefore had little influence on trait estimates.

Before imputation, maximum growth rates and phosphate affinities from Bruggeman (2011) were normalized to the same reference temperature and reference lighting period following the method described in Bruggeman (2011). Phosphate affinity data from Edwards et al. (2013) come from chemostat phosphate limitation experiments, to which parameters of Michaelis-Menten and Droop equations were fitted. Dividing the thus obtained phosphate uptake affinity by the minimum phosphate quota results in an affinity comparable to the affinity of the Monod equation.

To assess the sensitivity of our analyses to variability in taxonomic composition within classes and errors in trait assignment and taxonomy, we calculated for each year y estimates of community mean traits with two additional approaches. The first approach is based on 42-year average class mean traits weighted by the relative biovolumes of the taxonomic classes in year y:

$$CMT_1^*(y) = \exp\left(\sum_j^m \overline{\ln\left(GMT_j(y)\right)} \cdot g_j(y)\right) \tag{1}$$

where $GMT_j(y)$ is defined in SI 2, eq. 3. The second approach is based on the originally calculated class mean traits in year y weighted by the 42-year average relative biovolumes of the classes:

$$CMT_2^*(y) = \exp\left(\sum_j^m \ln\left(GMT_j(y)\right) \cdot \overline{g_j(y)}\right) \tag{2}$$

We used correlation analysis to compare $CMT_1^*(y)$ and $CMT_2^*(y)$, respectively, to the community mean traits as calculated with eq. 3 in the main text (Fig. S1). The community mean traits used in the main

text were consistently best approximated by $CMT_1^*(y)$, i.e. when using averaged class mean traits weighted by varying class relative biovolumes (88%, 81%, 92% explained variance for phosphate affinity, light affinity and maximum growth rate, respectively). In contrast, community mean traits based on $CMT_2^*(y)$ did, at best, weakly correlate with the trait values used in the main text (9% explained variance for light affinity) or were entirely uncorrelated (phosphate affinity and maximum growth rate).

These results illustrate that changes in community function largely arise due to changes in composition at the class level, rather than compositional changes within classes. Consequently, given that unmeasured trait values within the six most abundant taxa have been imputed at a taxonomic resolution a or below class level (with the exception of phosphate affinities of *Dinophyceae* spp.), we conclude that our analysis of community mean traits is insensitive to within-class variation in phylogeny, composition and function and that our imputation method therefore provides reliable results.

Note that the trait imputation approach used in this paper differs from an approach of PhyloPars (Bruggeman et al. 2009, Bruggeman 2011), which has been used to estimate missing traits in studies of seasonal dynamics of phytoplankton community mean traits including the one by Wentzky et al. (2020) which we briefly address in the discussion. This PhyloPars approach estimates missing trait values based on taxonomy and on fitted allometric scaling relationships between traits. While there are good reasons to believe that allometric trait scaling relationships could improve estimates of missing trait values, the assumption of trait scaling relationships should automatically produce trait-trait correlations among taxa for which traits have been estimated. Since the aim of our study was to search for possible correlations and tradeoffs between traits in the first place, we did not want to use an approach that could have biased analyses in favor of detecting trait-trait correlations. We therefore estimated missing traits based on taxonomy alone. In doing so, we relied strongly on the database of Bruggeman (2011), but we only used original source data and not the trait values estimated using allometric scaling.

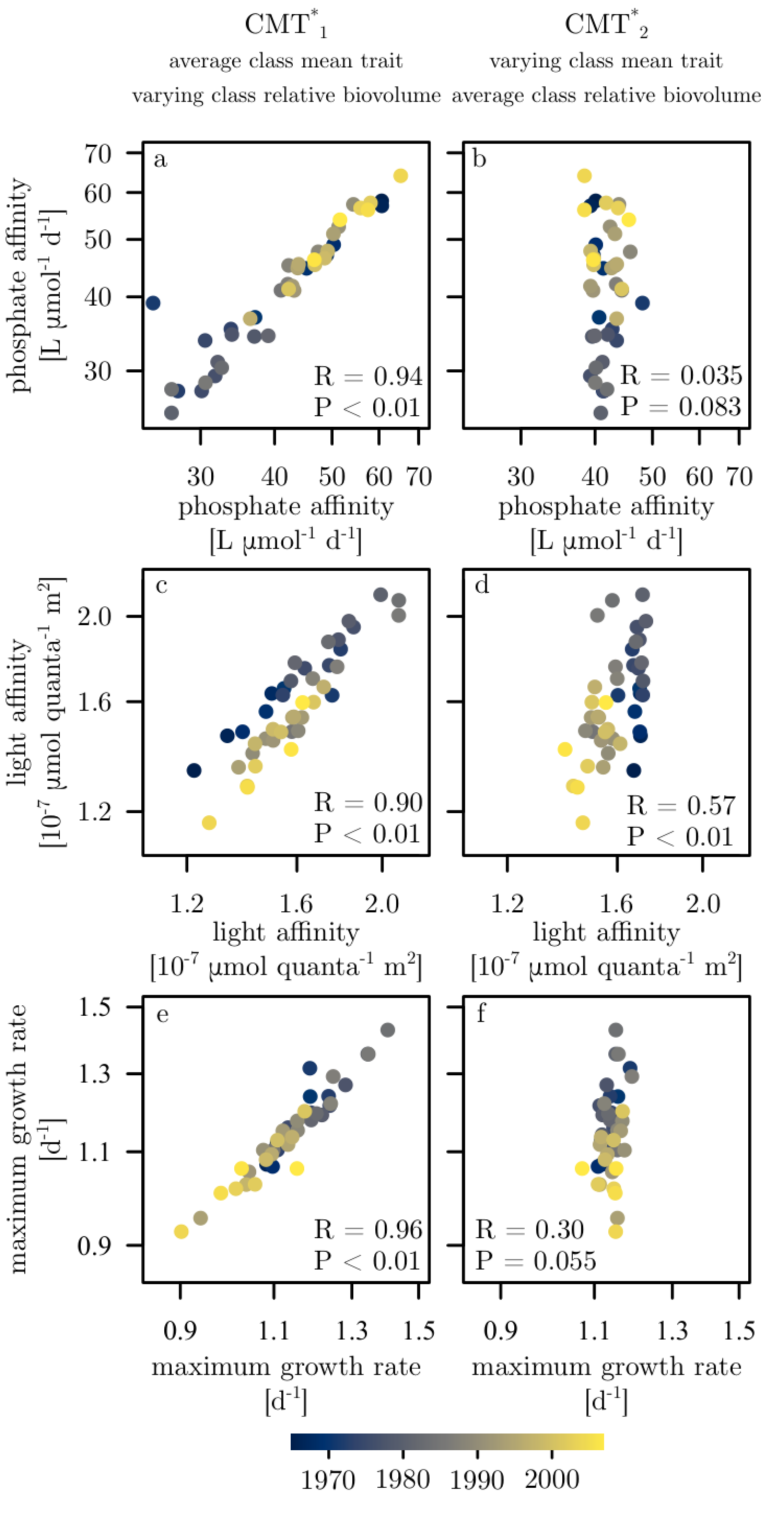

Fig. S1. Community mean phosphate affinity (a-b), light affinity (c-d) and maximum growth rate (e-f) calculated with 42-year average class mean traits weighted by relative biovolumes of the taxonomic classes (x-axes in the left column of panels) and with average class mean traits weighted by 42-year average class relative biovolumes (x-axes in the right column of panels), plotted against the corresponding community mean traits as calculated with eq. 3 in the main text. The color of the data points indicates time (starting in

1966 with dark blue and ending in 2007 with yellow, see legend at bottom of figure). R and P denote the Pearson correlation coefficient and probability, respectively.

SI 2 – class analyses

Relationship between annual mean relative class biovolume and TP_{mix}

The annual mean relative biovolume of the six most dominant classes were tested for correlation with TP_{mix} across the 42 study years. The classes *Cryptophyceae*, *Bacillariophyceae* and *Dinophyceae* were significantly anti-correlated to TP_{mix} (R_{pw} = -0.55, R_{pw} = -0.45 and R_{pw} = -0.62, respectively). Both *Chlorophyceae* and *Cyanophyceae* were positively correlated to TP_{mix} (R_{pw} = 0.70 and R_{pw} = 0.48, respectively). The relative biovolume of *Mediophyceae* was not correlated to TP_{mix}.

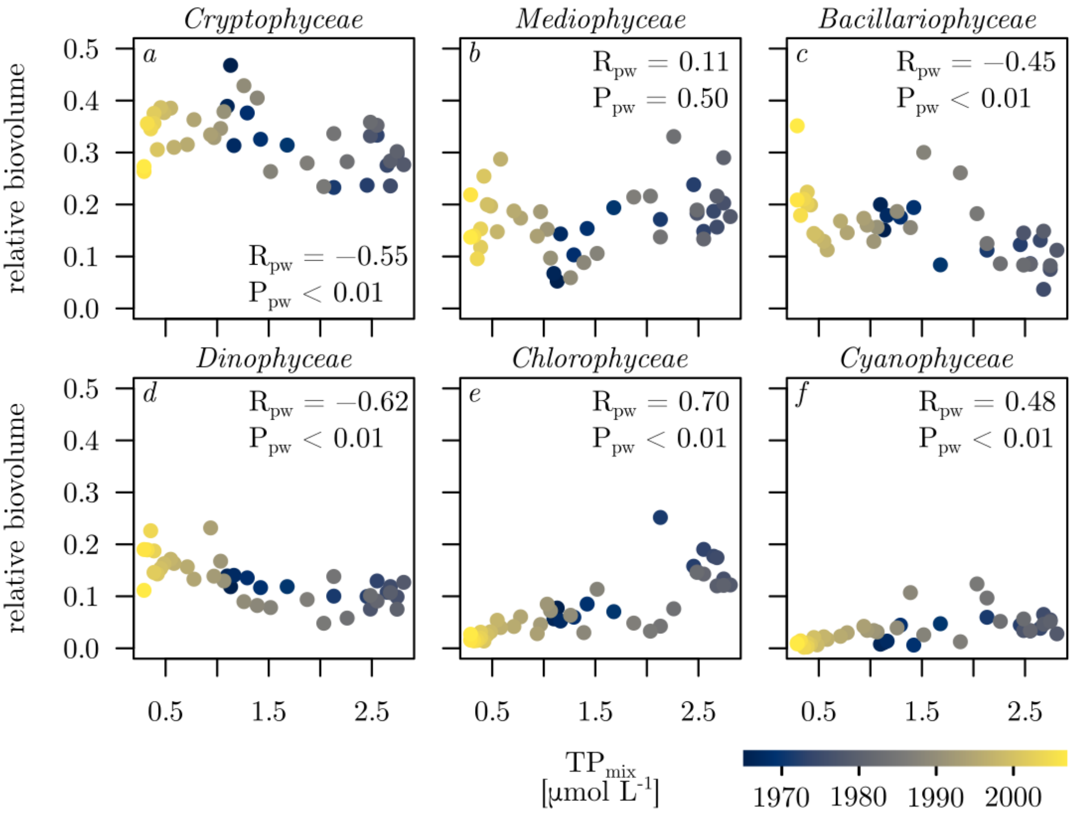

Figure S2. Annual mean relative biovolumes of the six most dominant classes plotted against TP_{mix} across the 42 study years. The color of the data points indicates time (starting in 1966 with dark blue and ending in 2007 with yellow, see legend in bottom-right corner). R_{pw} and P_{pw} represent the correlation coefficient and significance obtained through Prais-Winsten regression, respectively.

Class mean traits and their relationship to TP_{mix}

By renormalizing annual average relative biovolumes of species $b_i(y) : i \in \mathcal{I}_j$, where $\mathcal{I}_j$ is the set of indices corresponding to species in taxonomic class j, to the relative class biovolume $g_j(y)$, one can calculate class mean trait using

$$GMT_j(y) = \exp\left(\sum_{i \in \mathcal{I}_j} \frac{\ln(\tau_i) \cdot b_i(y)}{g_j(y)}\right) \tag{3}$$

Eq. 1 satisfies the condition that the community mean trait is the geometric average of the class mean trait weighted by the relative class abundance

$$CMT(y) = \exp\left(\sum_{j}^{m} \ln\left(GMT_j(y)\right) \cdot g_j(y)\right) \tag{4}$$

Class mean traits were calculated for the six most important classes. The rare classes were grouped together by appending their $\mathcal{I}_j$ and summing their $g_j(y)$ to obtain a single mean trait time series. Means and standard deviations are shown in Fig. S3 and their relationship to TP_{mix} is shown in Fig. S4. *Cryptophyceae* mean light affinity and maximum growth rate were significantly correlated and anti-correlated to TP_{mix}, respectively (R_{pw} = 0.77, P_{pw} < 0.01 and R_{pw} = -0.63, P_{pw} < 0.01). *Mediophyceae* mean phosphate affinity and maximum growth rate were significantly anti-correlated to TP_{mix} (R_{pw} = -0.75, P_{pw} < 0.01 and R_{pw} =). *Bacillariophyceae* mean phosphate affinity, light affinity and maximum growth rate were significantly correlated to TP_{mix} (R_{pw} = 0.38, P_{pw} = 0.012, R_{pw} = 0.8, P_{pw} < 0.01 and R_{pw} = 0.33, P_{pw} = 0.033). *Dinophyceae* mean maximum growth rate was significantly correlated to TP_{mix} (R_{pw} = 0.99, P_{pw} < 0.01) and lastly, *Cyanophyceae* mean phosphate affinity was significantly anti-correlated to TP_{mix}, respectively (R_{pw} = -0.33, P_{pw} = 0.033).

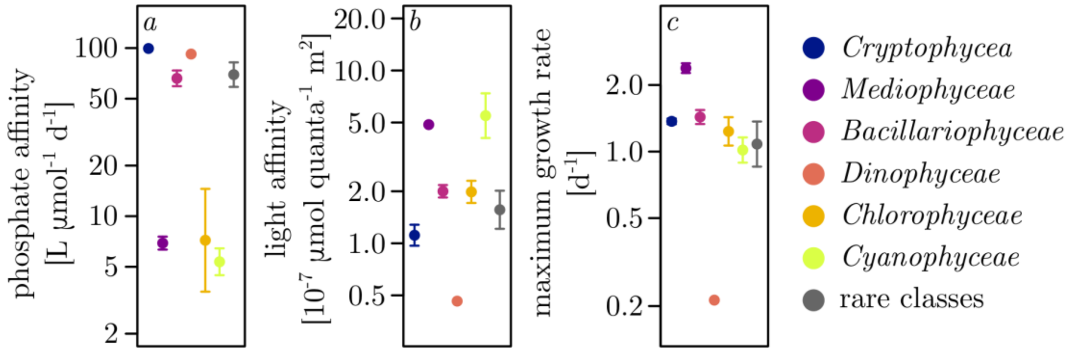

Figure S3. 42-year mean and standard deviation of class mean phosphate affinity (a), light affinity (b) and maximum growth rate (c). Circles indicate the mean and whiskers indicate the standard deviation. Colors identify the classes.

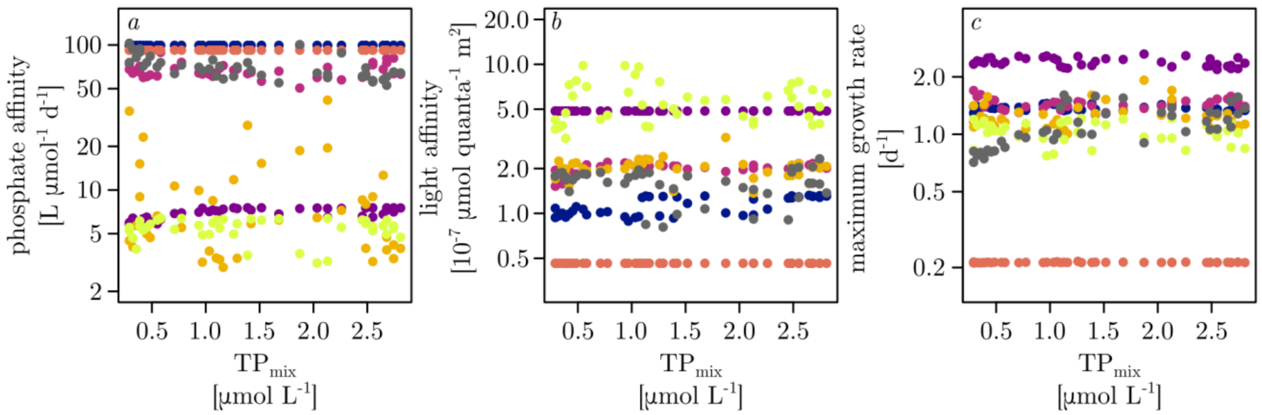

Figure S4. Relationship of class mean phosphate affinity (a), light affinity (b) and maximum growth rate (c) with TP_{mix}. Colors identify the classes (see legend Fig. S3).

Data analysis excluding Dinophyceae

Permutation tests revealed that the relationship between community mean maximum growth rate and TP_{mix} was sensitive to the low maximum growth rate and relative abundance of *Dinophyceae*. We therefore calculated $CMT_j'(y)$ of the community excluding the *Dinophyceae*, the results of which are presented in Fig S5. We first describe the general approach to this calculation and then present the results.

To obtain community mean trait values excluding species from taxonomic class j, we calculate the total biovolume of species not belonging to class j, $B_j'(t) = \sum_{i \in \mathcal{L}_j} B_i(t)$ where $\mathcal{L}_j \in \{1, \dots, n\} : \mathcal{L}_j \notin \mathcal{I}_j$. We may then obtain the relative biovolume of all species $i \in \mathcal{L}_j$ with $b_{ij}'(t) = B_i(t)/B_j'(t)$.

$b_{ij}'(t)$ was linearly interpolated to daily resolution and averaged over the 365/366 days of the respective year to obtain annual mean relative biovolumes $b_{ij}'(y)$. Then the community mean trait value excluding class j is the geometric mean of traits of species $i \in \mathcal{L}_j$, weighted by their annual mean biovolumes

$$CMT_j'(y) = \exp\left(\sum_{i \in \mathcal{L}_j} ln(\tau_i) \cdot b_{ij}'(y)\right) \tag{5}$$

where $CMT_j'(y)$ is the value of the community mean trait excluding class j in year y and τ_i is the trait value of species i.

When *Dinophyceae* were excluded from calculations, community mean phosphate affinity and light affinity shifted down and up, respectively compared to community means including *Dinophyceae* (Fig. S5a, d). Yet, the general patterns and respective negative and positive correlations with TP_{mix} and PC1 are robust against the exclusion of *Dinophyceae* from the community (Fig. S5b, c, e, f). In contrast, the temporal pattern in community mean maximum growth rate changed substantially and the positive correlation to TP_{mix} became insignificant when *Dinophyceae* were excluded from the analysis (Fig. S5g, h).

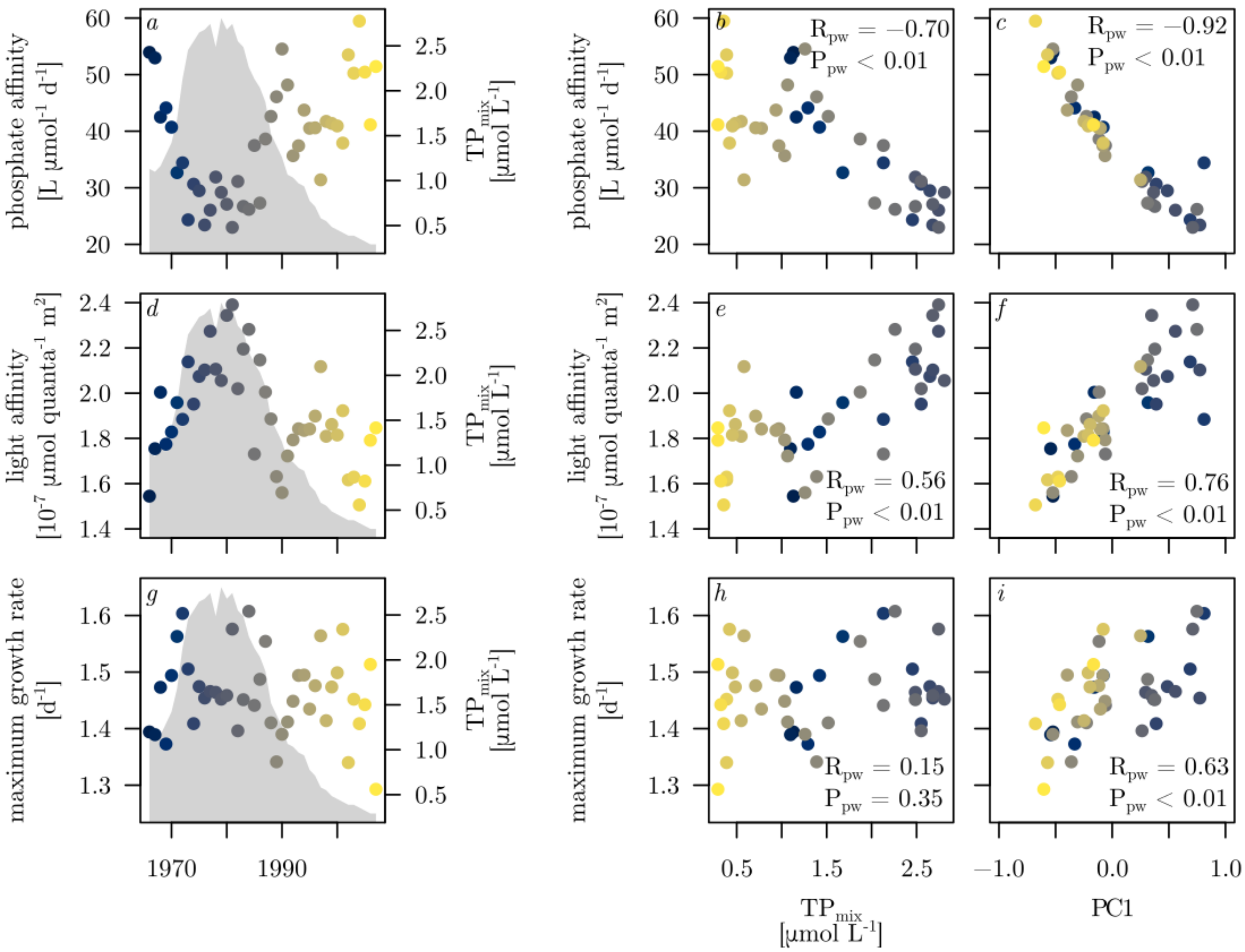

Figure S5. Community mean traits phosphate affinity (a-c), light affinity (d-f) and maximum growth rate (g-i), excluding Dinophyceae, plotted against study year (left panels), TP_{mix} (middle panels) and the first principal component from Fig. 2c in the main text (right panels). Grey shading in the left panels indicates TP_{mix}, and the color of the data points indicates the sampling year as in Fig. 2a (main text). R_{pw} and P_{pw} represent the correlation coefficient and significance obtained through Prais-Winsten regression, respectively. PC1 scores are scaled by a factor 5, as in Fig. 2.

After exclusion of *Dinophyceae* spp. from the analysis, pairwise correlations between all three traits could still be detected and the strength of which depended on the level of ecological and temporal integration. At the level of community membership (unweighted phenotypes), light affinity and phosphate affinity were negatively correlated, whereas pairwise correlations with maximum growth rate were insignificant (Fig. S6a-c). Relationships between traits at the level of community composition (biovolume weighted phenotypes) and the level of community mean trait dynamics were all significant and increased in strength with increasing level (Fig. S6d-i).

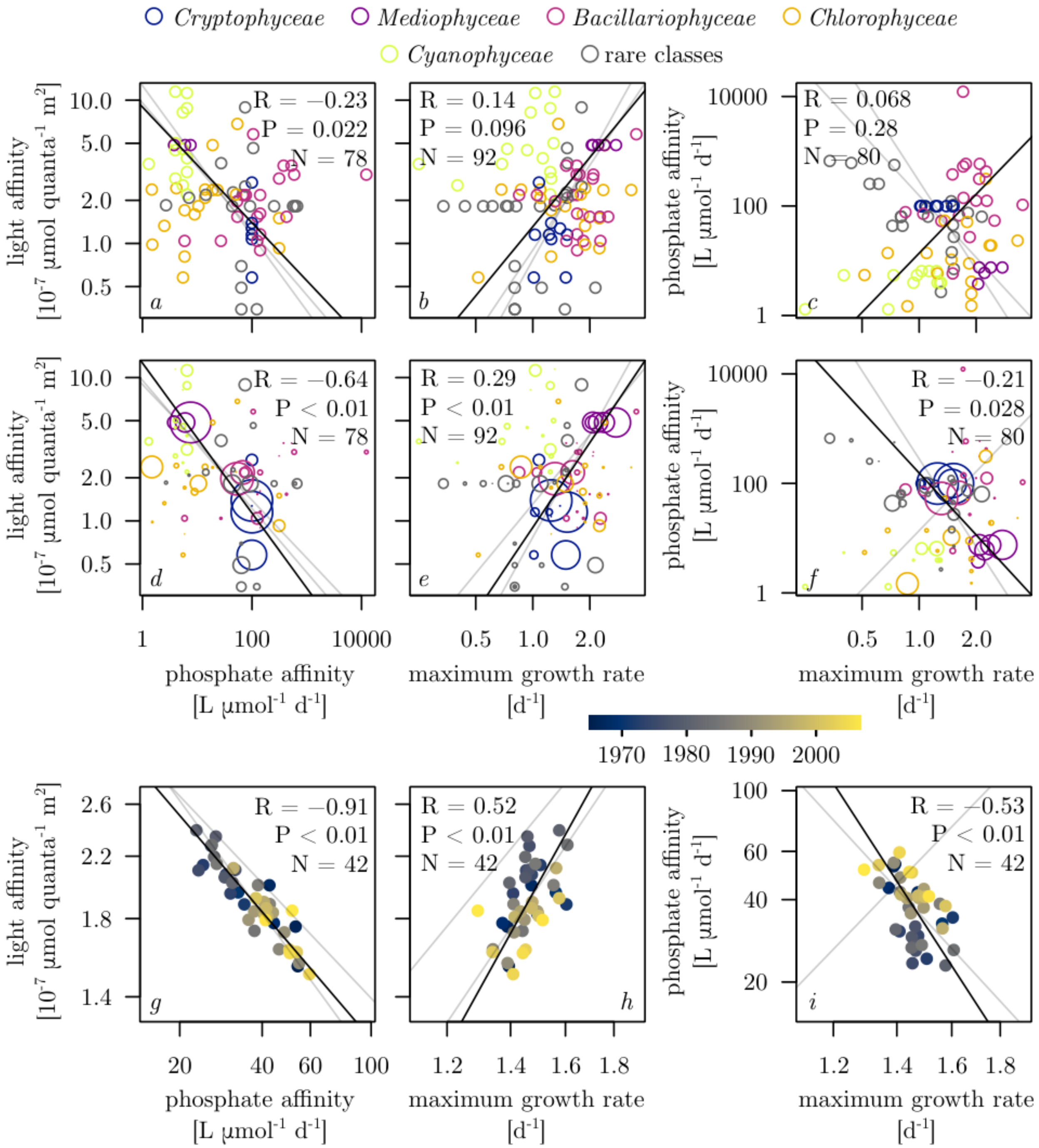

Figure S6. Pairwise plots of phosphate affinities, light affinities and maximum growth rates of Lake Constance phytoplankton phenotypes excluding those belonging to the class Dinophyceae. In panels a-c, circles represent the unweighted values of unique trait pairs (= phenotypes) that were present in the community. Panels d-f show the same data, but the area of the circles is weighted by the respective 42-year average relative biovolume corresponding to the unique trait pairs during the 42-year study period, $\overline{b_i}$*. Log-axes are not taken into consideration for determining the circle area and circles of the same size at different x or y values have the same 42-year average relative biovolume. Panels g-i show community mean traits for each of the 42 study years. Standardized major axis lines (in black) and statistical properties R and P were determined in standardized major axis fittings on log-transformed traits, the methods of which are*

described in SI 3. N indicates the number of unique trait pairs (a-f) or the number of years (g-i) used to determine P. To facilitate comparison of slopes and intercepts across the three levels of ecological and temporal integration, standardized major axes from the two panels above and/or below are always shown, but with increased transparency. Values of slope estimates and confidence intervals are shown in Fig. S7a-c. In panels a-f, data points are colored as in Fig. 2b (main text) to indicate the taxonomic classes to which the different trait pairs belong. In panels g-i, data points are colored as in Fig. 2a (main text) to indicate the sampling year.

We calculated slope estimates and confidence intervals (SI 3) and explained variances of log-transformed traits on the unweighted, weighted and community mean levels for both the data including and excluding trait data and relative biovolumes of species belonging to the class *Dinophyceae*. The results are shown in Fig. S7. Slope estimates across the three levels were consistently higher in standardized major axis fittings on light affinity and maximum growth rate when *Dinophyceae* were excluded. Slope estimates scattered strongly in SMA fittings on maximum growth rate and phosphate affinity. Explained variance consistently increased from the unweighted to unweighted to the biovolume-weighted to the community mean levels. Pairwise relationships at the unweighted, weighted and community mean level involving maximum growth rate were highly sensitive to the exclusion of *Dinophyceae* and consistently showed a reduced explained variance with respect to data including the *Dinophyceae*.

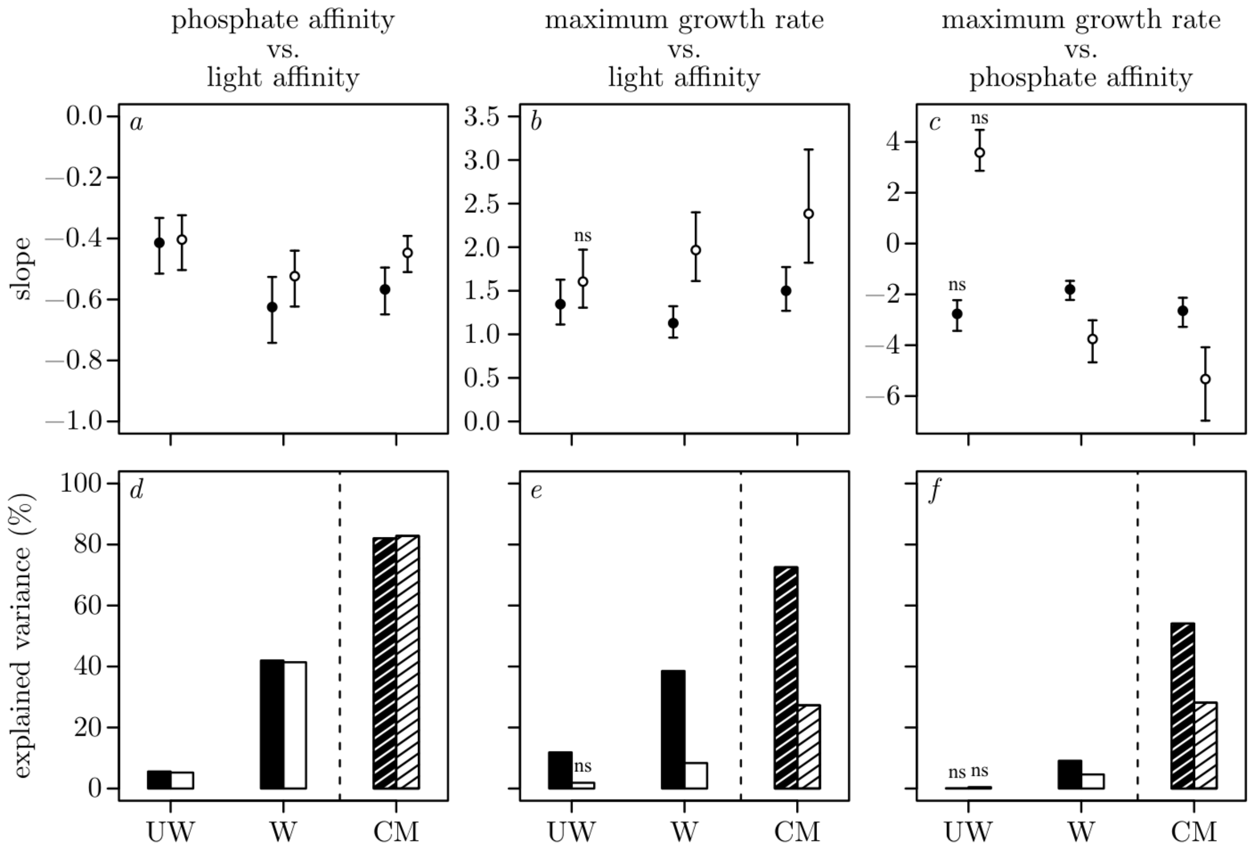

Figure S7. Slope estimates and 95% confidence intervals (a-c) and corresponding explained variances (d-f) of unweighted (UW), weighted (W) and community mean (CM) log-transformed trait pairs phosphate affinity and light affinity (a, d), maximum growth rate and light affinity (b, e) and maximum growth rate and phosphate affinity (c, f). Black points and bars result from analyses including Dinophyceae traits and relative biovolumes, white points and bars result from analyses excluding Dinophyceae traits and relative abundances (SI 3). 'ns' abbreviates not significant. The dashed line and hatch patterns are used to

communicate that explained variances of log-transformed community mean traits should not be directly compared to explained variances of unweighted and weighted log-transformed trait data. Community mean traits have a temporal component whereas unweighted and weighted species traits do not. The averaging step used to obtain community mean traits likely introduces additional explained variance.

We also tested whether the mixotrophic classes *Chrysophyceae*, *Synurophyceae* and *Coccolithophyceae* influenced the community mean maximum growth rate-TP_{mix} correlation. Community mean trait values excluding multiple classes were calculated by substituting $\mathcal{I}_j$ in calculations presented in this section with the set of indices corresponding to species belonging to any of the excluded classes. Exclusion of the classes *Chrysophyceae*, *Synurophyceae* and *Coccolithophyceae* did not substantially affect the relationship between community mean maximum growth rate and TP_{mix} (Fig. S8).

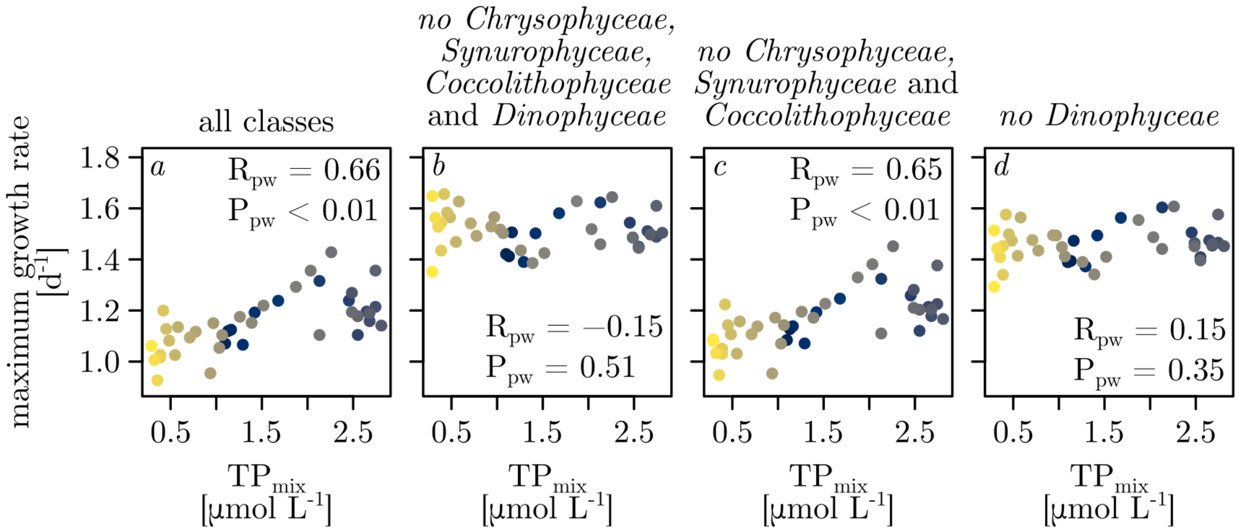

Figure S8. Community mean maximum growth rate calculated with (a) all data, (b) data excluding all potentially important mixotrophic classes, (c) data excluding Chrysophyceae spp., Synurophyceae spp. and Coccolithophyceae spp. and (d) data excluding Dinophyceae spp., plotted against TP_{mix}.

SI 3 – Standardized major axis fitting

In this work, we employ standardized major axis (SMA) fitting methods described in Warton et al. (2006). To summarize, the SMA is fitted to log-transformed values X and Y of traits p and q, giving the following relationship:

$$Y = \hat{\alpha} + \hat{\beta} X \tag{6.1}$$

$$q = e^{\hat{\alpha}} p^{\hat{\beta}} \tag{6.2}$$

where $\hat{\alpha}$ is the intercept estimate and $\hat{\beta}$ is the slope estimate of the SMA. $\hat{\beta}$ is the ratio of the sample standard deviation of Y (s_y) to the sample standard deviation of X (s_x), multiplied by the sign of the sample covariance of X and Y (s_{xy}):

$$\hat{\beta} = \mathrm{sign}(s_{xy})\frac{s_y}{s_x} \tag{7}$$

and $\hat{\alpha}$ can be obtained by subtracting the slope multiplied by the sample mean of X ($\bar{x}$) from the sample mean of Y ($\bar{y}$):

$$\hat{\alpha} = \bar{y} - \hat{\beta}\bar{x} \tag{8}$$

The confidence interval C_p for $\hat{\beta}$ is calculated as:

$$C_p = \hat{\beta}\left(\sqrt{B+1} \pm \sqrt{B}\right) \tag{9.1}$$

where

$$B = \frac{1-R^2}{N-2} f_{1-p,1,N-2} \tag{9.2}$$

and R is the sample Pearson correlation coefficient, N is the number of observation pairs and $f_{1-p,1,N-2}$ is the critical value of the f distribution at confidence level p and degrees of freedom $1,\ N-2$.

Eq. 7-9.2 were used to obtain SMAs in Fig. 4 (main text) and Fig. S6. To avoid bias introduced due to estimated trait pair replicates, we let N be the number of unique trait pairs in the biovolume-weighted and unweighted trait correlations. In the last row of Fig. 4 (main text) and Fig. S6, where SMAs are fitted on community mean traits, N corresponds to the number of years. N is shown in each of the panels in Fig. 4 (main text) and Fig. S6.

Warton et al. (2006) do not provide a method for weighted SMA fitting. For the biovolume-weighted SMA, we replicated the observation pairs of X and Y, where the number of replications is determined by the 42-year average relative biovolume of the respective species multiplied by 10^4 and rounded to the nearest integer. We then computed Eq. 7-9.2.

Relative biovolume along the major axis

Residual values (U), i.e. the scores along the minor axis are, just as in the linear regression, the difference between $Y$and the prediction of Y:

$$U = Y - \hat{\alpha} - \hat{\beta}X \tag{10.1}$$

The fitted values (V), i.e. the scores along the major axis of the covariance ellipse are, in case of SMA fitting, the sum Y and the prediction of Y:

$$V = Y + \hat{\alpha} + \hat{\beta}X$$

(10.2)

Any pair of U and V coordinates can be transformed back to X and Y coordinates with the following relationships:

$$Y = \frac{V + U}{2}$$

(11.1)

and

$$X = \frac{V - U}{2\hat{\beta}} - \frac{\hat{\alpha}}{\hat{\beta}}$$

(11.2)

The biovolume distribution along the SMA can be summarized by the 0th, 25th, 50th, 75th and 100th percentiles of the fitted values Q_v, weighted by the relative biovolume of the corresponding species. These percentiles can be transformed to their respective X and Y coordinates Q_x and Q_y by substituting V with Q_v and U with 0 in Eq. 11.1 and Eq. 11.2.

$$Q_y = \frac{Q_v}{2}$$

(12.1)

and

$$Q_x = \frac{Q_v}{2\hat{\beta}} - \frac{\hat{\alpha}}{\hat{\beta}}$$

(12.2)

Warton et al. (2006) omit $\hat{\alpha}$ in Eq. 10.1 and Eq. 10.2. $\hat{\alpha}$ only translates U and V and does not affect their orthogonality. In that case, one would substitute U and V with $U - \hat{\alpha}$ and $V + \hat{\alpha}$ in Eq. 11.2 and Q_v with $Q_v + \hat{\alpha}$ in Eq. 12.2.

Any $\hat{\alpha}$ and $\hat{\beta}$ satisfying Eq. 8 can be used to obtain V scores and biovolume weighted percentiles Q_v in a desired direction (except $\hat{\beta} = 0$ and $\beta = \pm\infty$, which correspond to the biovolume distribution in X and Y, respectively). To allow comparisons of biovolume distributions in different periods, Q_v of each period must be determined with the same $\hat{\alpha}$ and $\hat{\beta}$. In Fig. 5a-b (main text), the distribution of average relative biovolumes from 5 eutrophic years (1980 – 1984) and 5 oligotrophic years (2002 – 2006) were shown along the SMA fitted to values of phosphate affinity and light affinity weighted by 42-year average relative biovolume (Fig. 4d, main text).
